# Supplementary material for: Comparative Genomics and Pan-Genome Driven Prediction of a Reduced Genome of Akkermansia muciniphila
Source: Microorganisms. 2022 Jul 4;10(7):1350. doi: 10.3390/microorganisms10071350 (PMC9315967; doi:10.3390/microorganisms10071350)
Supplement: Supplementary file 1 [file microorganisms-10-01350-s001.zip › microorganisms-1747541-supplementary/supplementary/Supplementary figures.pdf]

Supplementary figures

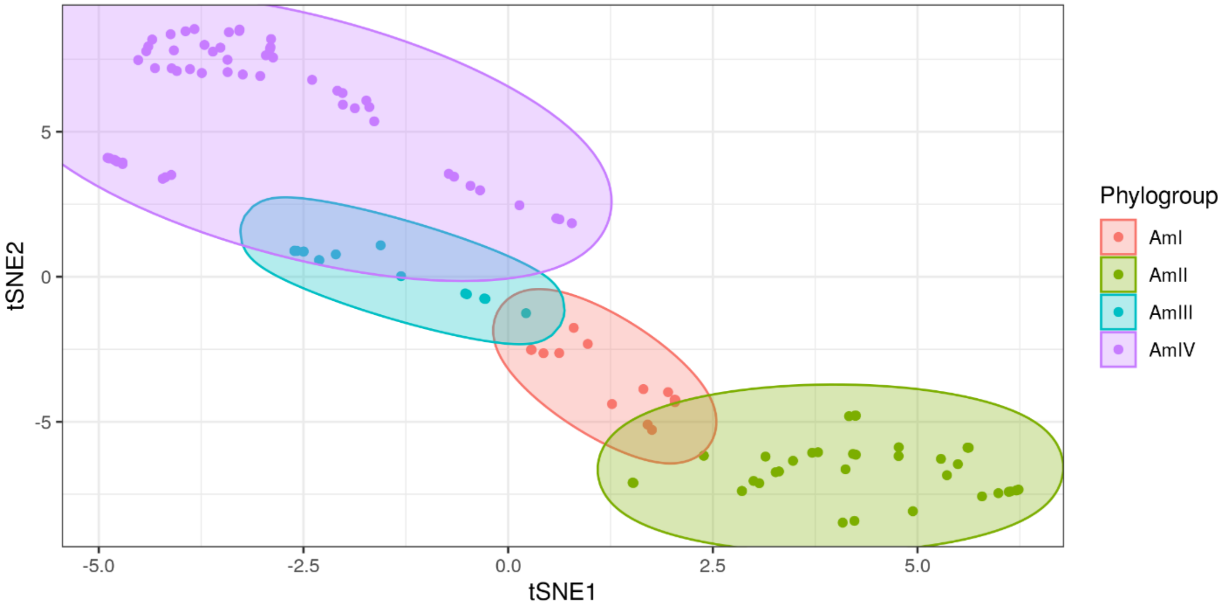

Figure S1: tSNE plot of gene presence absence colored by different phylogroups. Strains are clearly clustered in four phylogroups.

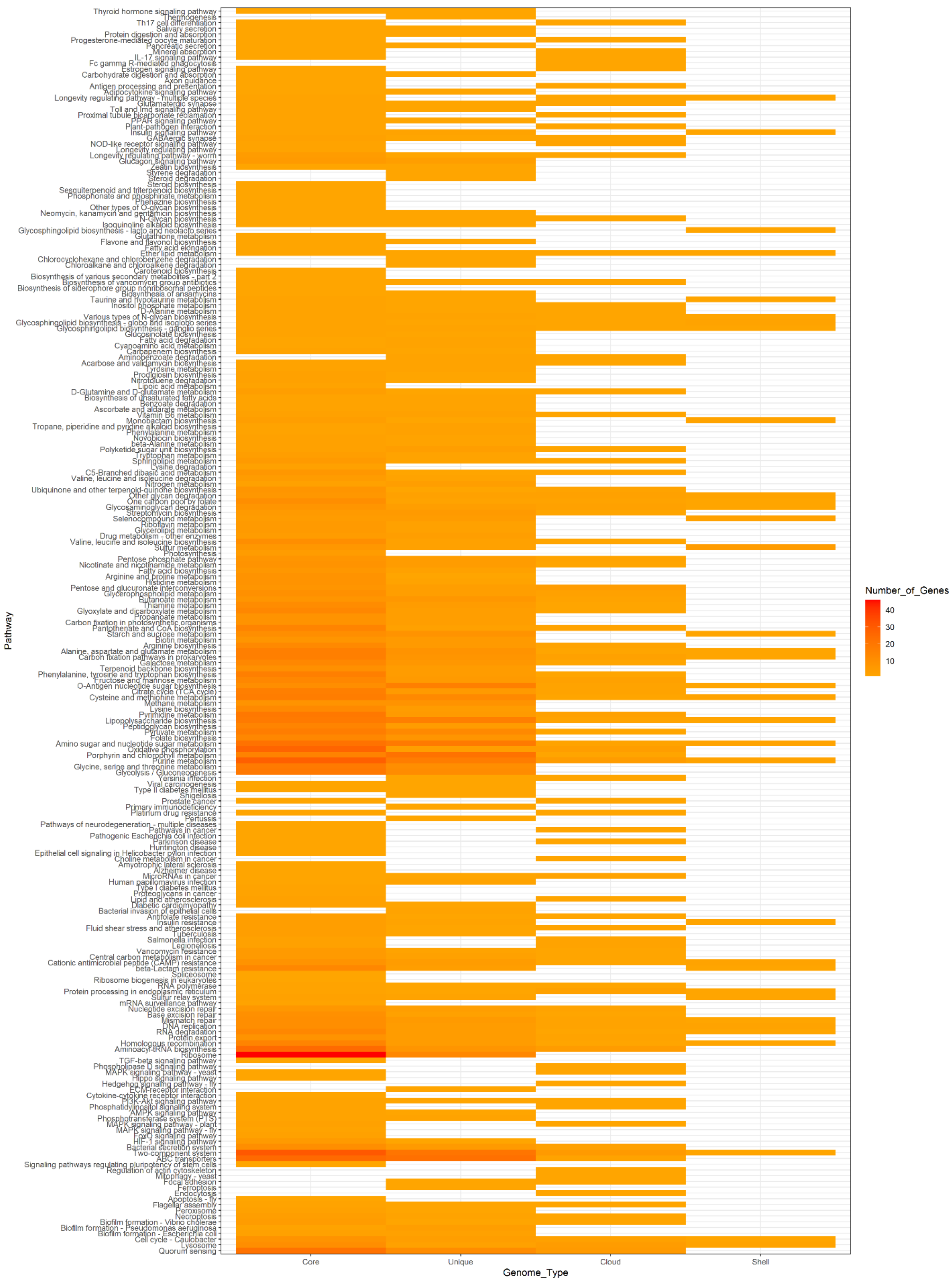
Pathway
Thyroid hormone signaling pathway
Thermogenesis
Th17 cell differentiation
Salivary secretion
Protein digestion and absorption
Progesterone-mediated oocyte maturation
Pancreatic secretion
Mineral absorption
IL-17 signaling pathway
Fc gamma R-mediated phagocytosis
Estrogen signaling pathway
Carbohydrate digestion and absorption
Axon guidance
Antigen processing and presentation
Adipocytokine signaling pathway
Longevity regulating pathway - multiple species
Glutamatergic synapse
Toll and Imd signaling pathway
Proximal tubule bicarbonate reclamation
PPAR signaling pathway
Plant-pathogen interaction
Insulin signaling pathway
GABAergic synapse
NOD-like receptor signaling pathway
Longevity regulating pathway
Longevity regulating pathway - worm
Glucagon signaling pathway
Zeatin biosynthesis
Styrene degradation
Steroid degradation
Steroid biosynthesis
Sesquiterpenoid and triterpenoid biosynthesis
Phosphonate and phosphinate metabolism
Phenazine biosynthesis
Other types of O-glycan biosynthesis
Neomycin, kanamycin and gentamicin biosynthesis
N-Glycan biosynthesis
Isoquinoline alkaloid biosynthesis
Glycosphingolipid biosynthesis - lacto and neolacto series
Glutathione metabolism
Flavone and flavonol biosynthesis
Fatty acid elongation
Ether lipid metabolism
Chlorocyclohexane and chlorobenzene degradation
Chloroalkane and chloroalkene degradation
Carotenoid biosynthesis
Biosynthesis of various secondary metabolites - part 2
Biosynthesis of vancomycin group antibiotics
Biosynthesis of siderophore group nonribosomal peptides
Biosynthesis of ansamycins
Taurine and hypotaurine metabolism
Inositol phosphate metabolism
D-Alanine metabolism
Various types of N-glycan biosynthesis
Glycosphingolipid biosynthesis - globo and isoglobo series
Glycosphingolipid biosynthesis - ganglio series
Glucosinolate biosynthesis
Fatty acid degradation
Cyanoamino acid metabolism
Carbapenem biosynthesis
Aminobenzoate degradation
Acarbose and validamycin biosynthesis
Tyrosine metabolism
Prodigiosin biosynthesis
Nitrotoluene degradation
Lipoic acid metabolism
D-Glutamine and D-glutamate metabolism
Biosynthesis of unsaturated fatty acids
Benzoate degradation
Ascorbate and aldarate metabolism
Vitamin B6 metabolism
Monobactam biosynthesis
Tropane, piperidine and pyridine alkaloid biosynthesis
Phenylalanine metabolism
Novobiocin biosynthesis
beta-Alanine metabolism
Polyketide sugar unit biosynthesis
Tryptophan metabolism
Sphingolipid metabolism
Lysine degradation
C5-Branched dibasic acid metabolism
Valine, leucine and isoleucine degradation
Nitrogen metabolism
Ubiquinone and other terpenoid-quinone biosynthesis
Other glycan degradation
One carbon pool by folate
Glycosaminoglycan degradation
Streptomycin biosynthesis
Selenocompound metabolism
Riboflavin metabolism
Glycerolipid metabolism
Drug metabolism - other enzymes
Valine, leucine and isoleucine biosynthesis
Sulfur metabolism
Photosynthesis
Pentose phosphate pathway
Nicotinate and nicotinamide metabolism
Fatty acid biosynthesis
Arginine and proline metabolism
Histidine metabolism
Pentose and glucuronate interconversions
Glycerophospholipid metabolism
Butanoate metabolism
Thiamine metabolism
Glyoxylate and dicarboxylate metabolism
Propanoate metabolism
Carbon fixation in photosynthetic organisms
Pantothenate and CoA biosynthesis
Starch and sucrose metabolism
Biotin metabolism
Arginine biosynthesis
Alanine, aspartate and glutamate metabolism
Carbon fixation pathways in prokaryotes
Galactose metabolism
Terpenoid backbone biosynthesis
Phenylalanine, tyrosine and tryptophan biosynthesis
Fructose and mannose metabolism
O-Antigen nucleotide sugar biosynthesis
Citrate cycle (TCA cycle)
Cysteine and methionine metabolism
Methane metabolism
Lysine biosynthesis
Pyrimidine metabolism
Lipopolysaccharide biosynthesis
Peptidoglycan biosynthesis
Pyruvate metabolism
Folate biosynthesis
Amino sugar and nucleotide sugar metabolism
Oxidative phosphorylation
Porphyrin and chlorophyll metabolism
Purine metabolism
Glycine, serine and threonine metabolism
Glycolysis / Gluconeogenesis
Yersinia infection
Viral carcinogenesis
Type II diabetes mellitus
Shigellosis
Prostate cancer
Primary immunodeficiency
Platinum drug resistance
Pertussis
Pathways of neurodegeneration - multiple diseases
Pathways in cancer
Pathogenic Escherichia coli infection
Parkinson disease
Huntington disease
Epithelial cell signaling in Helicobacter pylori infection
Choline metabolism in cancer
Amyotrophic lateral sclerosis
Alzheimer disease
MicroRNAs in cancer
Human papillomavirus infection
Type I diabetes mellitus
Proteoglycans in cancer
Lipid and atherosclerosis
Diabetic cardiomyopathy
Bacterial invasion of epithelial cells
Antifolate resistance
Insulin resistance
Fluid shear stress and atherosclerosis
Tuberculosis
Salmonella infection
Legionellosis
Vancomycin resistance
Central carbon metabolism in cancer
Cationic antimicrobial peptide (CAMP) resistance
beta-Lactam resistance
Spliceosome
Ribosome biogenesis in eukaryotes
RNA polymerase
Protein processing in endoplasmic reticulum
Sulfur relay system
mRNA surveillance pathway
Nucleotide excision repair
Base excision repair
Mismatch repair
DNA replication
RNA degradation
Protein export
Homologous recombination
Aminoacyl-tRNA biosynthesis
Ribosome
TGF-beta signaling pathway
Phospholipase D signaling pathway
MAPK signaling pathway - yeast
Hippo signaling pathway
Hedgehog signaling pathway - fly
ECM-receptor interaction
Cytokine-cytokine receptor interaction
PI3K-Akt signaling pathway
Phosphatidylinositol signaling system
AMPK signaling pathway
Phosphotransferase system (PTS)
MAPK signaling pathway - plant
MAPK signaling pathway - fly
FoxO signaling pathway
HIF-1 signaling pathway
Bacterial secretion system
Two-component system
ABC transporters
Signaling pathways regulating pluripotency of stem cells
Regulation of actin cytoskeleton
Mitophagy - yeast
Focal adhesion
Ferroptosis
Endocytosis
Apoptosis - fly
Flagellar assembly
Peroxisome
Necroptosis
Biofilm formation - Vibrio cholerae
Biofilm formation - Pseudomonas aeruginosa
Biofilm formation - Escherichia coli
Cell cycle - Caulobacter
Lysosome
Quorum sensing
Core
Unique
Cloud
Shell
Genome_Type
Number_of_Genes
40
30
20
10

Figure S2: Comparison of the KEGG pathways across different section of the pan genome (Core, Unique, Cloud and Shell). The orange tile represents the presence of a genes while darker (redish) colors represents high number of genes in the corresponding category.
